# Inhibition of IRS-1 by hepatitis C virus infection leads to insulin resistance in a PTEN-dependent manner

**DOI:** 10.1186/s12985-015-0241-4

**Published:** 2015-02-03

**Authors:** Ting-ting Gao, Zhao-ling Qin, Hao Ren, Ping Zhao, Zhong-tian Qi

**Affiliations:** Department of Microbiology, Shanghai Key Laboratory of Medical Biodefense, Second Military Medical University, Shanghai, 200433 China

**Keywords:** Hepatitis C virus, Insulin resistance, IRS-1, pIRS-1 Ser307, PTEN

## Abstract

**Background:**

Hepatitis C virus (HCV) infection was recently recognized as an independent risk factor for insulin resistance (IR), the onset phase of type 2 diabetes mellitus (T2DM). Phosphatase and tensin homolog deleted on chromosome 10 (PTEN) negatively regulates PI3K/Akt signaling pathway, which is critical for IR development and progression of cirrhosis to hepatocellular carcinoma (HCC). Here, we investigate the role of PTEN in HCV-associated IR and explored the mechanisms by which HCV regulates PTEN.

**Methods:**

Western blotting was used to detect the levels of insulin signaling pathway components, including insulin receptor substrate-1 (IRS-1), phosphorylated IRS-1 (pIRS-1) at serine 307 (Ser307), both phosphorylated Akt (pAkt) and total Akt. A time-course experiment measuring activation of the insulin signaling pathway was performed to assess the effect of HCV infection on insulin sensitivity by examining the phosphorylation levels of Akt and GSK3β, a downstream target of Akt. Huh7.5.1 cells were transduced with a lentiviral vector expressing PTEN or PTEN shRNA, and IRS-1 and pIRS-1 (Ser307) levels were determined in both HCV-infected and uninfected cells. The pc-JFH1-core plasmid was constructed to explore the underlying mechanisms by which HCV regulated PTEN and therefore IRS-1 levels.

**Results:**

HCV infection inhibited the insulin signaling pathway by reducing the levels of IRS-1 and pAkt/Akt while increasing phosphorylation of IRS-1 Ser307. In addition, HCV infection decreased the sensitivity to insulin-induced stimulation by inhibiting Akt and GSK3β phosphorylation. Furthermore, PTEN mRNA and protein levels were reduced upon HCV infection as well as transfection with the pc-JFH1-core plasmid. The reduction in IRS-1 level observed in HCV-infected cells was rescued to a limited extent by overexpression of PTEN, which in turn slightly reduced pIRS-1 (Ser307) level. In contrast, IRS-1 level were significantly decreased and phosphorylation of IRS-1 at Ser-307 was strongly enhanced by PTEN knockdown, suggesting that both reduction in IRS-1 level and increase in IRS-1 phosphorylation at Ser307 upon HCV infection occurred in a PTEN-dependent manner.

**Conclusions:**

HCV infection suppresses the insulin signaling pathway and promotes IR by repressing PTEN, subsequently leading to decreased levels of IRS-1 and increased levels of pIRS-1 at Ser307. The findings provide new insight on the mechanism of HCV-associated IR.

## Introduction

HCV infection is prevalent in 1–3% of the global population and is a primary cause of liver diseases such as chronic hepatitis, steatosis, cirrhosis, and hepatocellular carcinoma (HCC) [1,2]. Chronic HCV infection is also associated with a multifaceted disorder correlating with various metabolic disorders, including insulin resistance (IR), type 2 diabetes mellitus (T2DM), lipid metabolism, obesity and liver steatosis [2,3]. IR is a systemic pre-diabetic disorder wherein an increased level of insulin needs to maintain normal glucose levels in peripheral tissues, especially in muscle and adipose tissue [4]. Previous studies have shown that IR is the major factor leading to hepatic steatosis and fibrosis, weakened host immune response to viral infection, and is involved in the development of HCC [5-7].

Recently, more studies have revealed that HCV infection appears to interfere with glucose homeostasis and ultimately induce T2DM [8]. Signaling events triggered by insulin stimulation are involved in the molecular mechanisms of IR. Binding of insulin to its receptor phosphorylates insulin receptor substrate 1/2 (IRS-1/2), which in turn activates the PI3K/Akt signaling pathway and ultimately leads to an increase in glucose uptake and glycogen synthesis [9]. Although the precise mechanisms underlying HCV-mediated IR remain poorly understood, several hypotheses have been proposed. During HCV infection, tumor necrosis factor (TNF)-α system is activated and interleukin-6 levels are increased, which disturb tyrosine phosphorylation of IRS-1. Suppressor of cytokine signaling-3 (SOCS-3) level was subsequently increased to promote proteasomal degradation of IRS-1/2. Moreover, HCV infection eventually leads to liver steatosis and fibrosis, increased oxidative stress and peroxidation, all of which trigger a cascade of inflammatory responses, thus contributing to the development of IR [9-11].

Phosphatase and tensin homolog deleted on chromosome 10 (PTEN) is first identified as a tumor suppressor that dephosphorylates both proteins and phosphoinositides [12]. However, whether PTEN plays a role in regulation of the insulin signaling pathway remains to be clarified. Vinciguerra M et al*.* showed that PTEN deletion of liver tissue led to enhanced peripheral glucose metabolism in mice [13]. Hypersensitivity to insulin of the results was consistent with the fact that PTEN might negatively regulate peripheral insulin sensitivity. Paradoxically, they also found that free fatty acid-mediated PTEN down-regulation caused resistance to some of the insulin metabolic effects in hepatoma HepG2 cells by decreasing phosphorylation of insulin receptors and subsequent IRS-1 expression [14]. Therefore, further studies are needed to clarify whether PTEN down-regulation in HCV-infected hepatocytes is also a causal factor for IR. To determine whether alterations in PTEN expression/activity in human hepatocytes were implicated in the development of IR during HCV infection, we investigated its expression pattern in HCV-infected cells and its impact on the modulation of IRS-1. We found that HCV infection down-regulates PTEN expression and conversely increases phosphorylation of IRS-1 at Ser307, which subsequently impairs the PI3K/Akt signaling pathway, leading to IR. These results provide new insights into the mechanisms of HCV-associated IR.

## Results

### HCV infection inhibits the insulin signaling pathway

**Figure 1:**
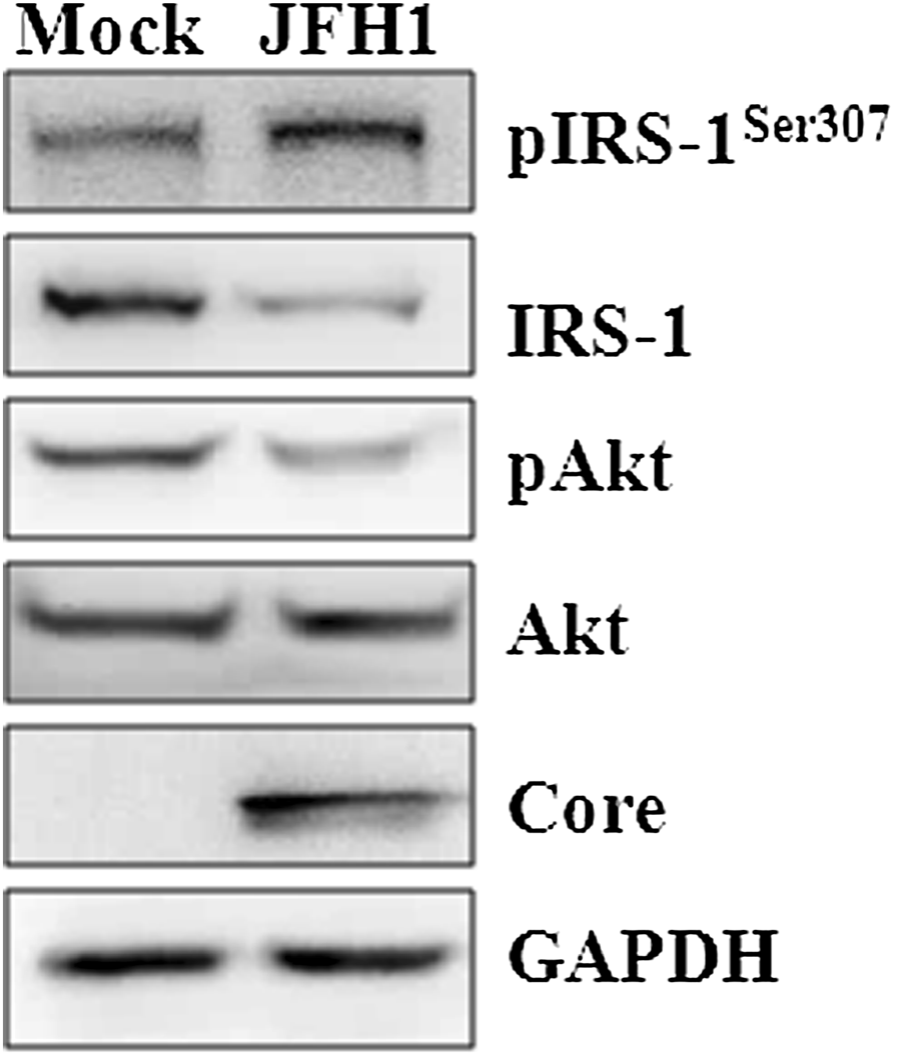
**Effect of HCV infection on the insulin signaling pathway.** Equal amounts of cellular lysates from uninfected and HCV-infected Huh7.5.1 cells at a M.O.I of 2 for 48 h were subjected to western blot analysis using anti-pIRS-1^Ser307^, IRS-1, pAkt, Akt, and core-specific antibodies. Anti-GAPDH antibody was used as an internal control to verify protein loading.

IRS-1, an adaptor protein for the insulin signaling pathway, undergoes proteasomal degradation and post-translational modification to arrive at a balance between its Tyr/Ser phosphorylation [3,4]. To determine the status of the insulin signaling pathway in HCV-infected cells, we examined the levels of IRS-1 and its Ser307-phosphorylated form in Huh7.5.1 cells infected with JFH1-based HCVcc. The phosphorylation of IRS-1 at Ser307 was remarkably increased, and both IRS-1 protein and pAkt/Akt levels were decreased (Figure 1), indicating that the PI3K/Akt signaling pathway was also attenuated in the course of HCV infection. These results suggest that HCV infection impairs the insulin signaling pathway by decreasing IRS-1 level and therefore PI3K/Akt signaling pathway.

### HCV infection decreases sensitivity of Huh7.5.1 cells to exogenous insulin

Activated Akt can induce phosphorylation of glycogen synthase kinase 3β (GSK3β), a key component of glycogen synthesis, at the serine residues and thus inactivate its kinase activity, which leads to glycogen production by activation of glycogen synthase [15]. To assess the effect of HCV infection on insulin sensitivity, we examined the phosphorylation status of Akt and GSK3β induced by insulin stimulation. We performed a time-course experiment in uninfected Huh7.5.1 cells, which showed that phosphorylation of Akt at Ser473 and GSK3β at Ser9 reached a maximum level at 30 min after stimulation with 100 nM insulin. Conversely, infected cells that were stimulated with the same concentration of insulin showed attenuated phosphorylation of Akt and GSK3β at the same serine residues. These results showed that HCV 2a induced insulin sensitivity by decreasing phosphorylation of Akt at Ser473 and GSK3β at Ser9.

### Down-regulation of IRS-1 and up-regulation of pIRS-1 at Ser307 upon HCV 2a infection is PTEN-dependent

**Figure 2:**
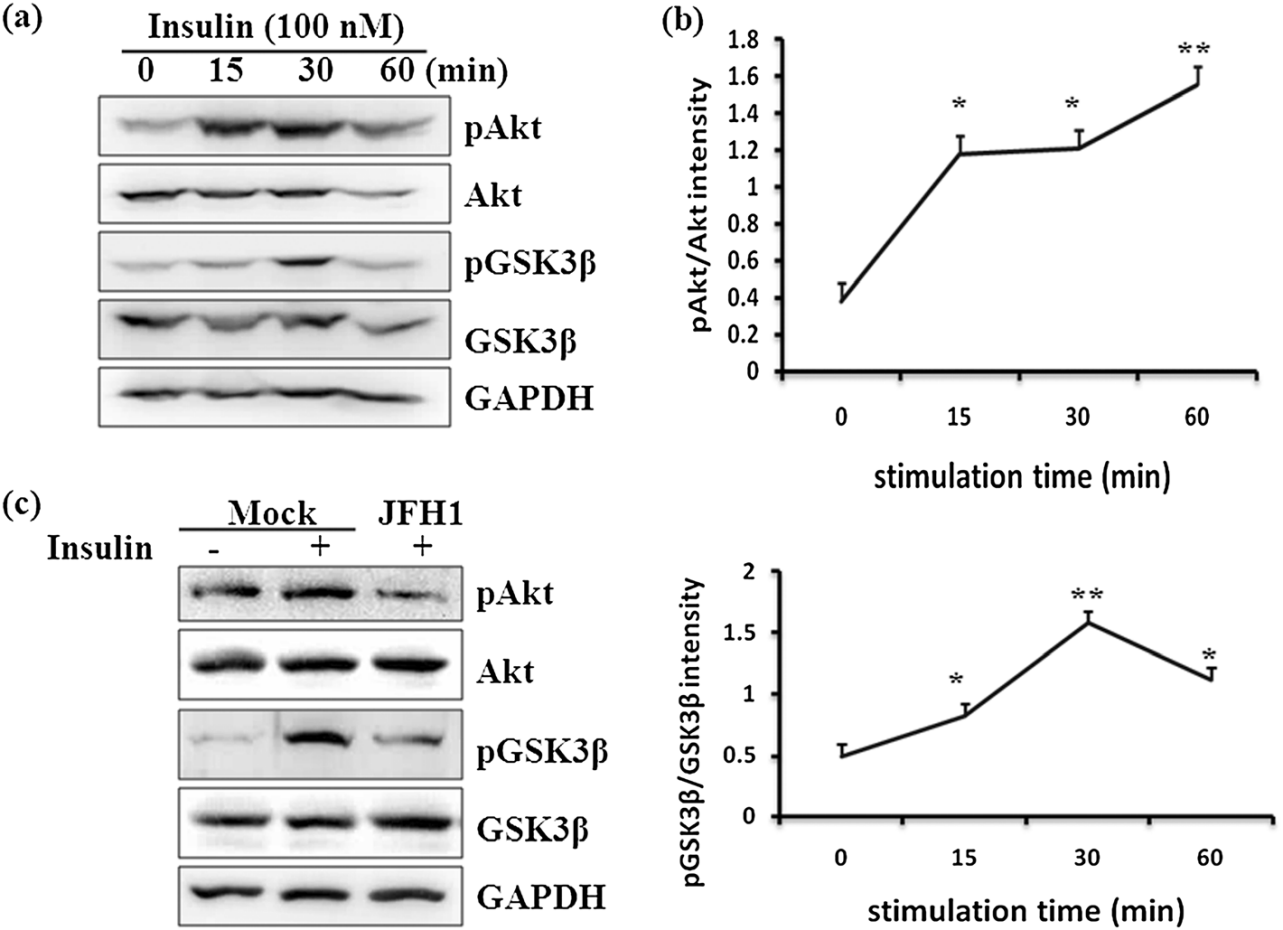
**Effect of HCV infection on insulin-induced phosphorylation of Akt and GSK3β.** Huh7.5.1 cells were seeded in a 24-well plate and subjected to serum-free incubation for 20 h, followed by insulin stimulation for the indicated time (0, 15, 30, and 60 min). Western blotting with indicated antibodies revealed a time-dependent change in the phosphorylation of Akt **(a)** and GSK3β **(b)**. Furthermore, Huh7.5.1 cells and HCV-infected cells were stimulated with 100 nM insulin for 30 min or left untreated. Extracted proteins were analyzed with antibodies against pAkt, Akt, pGSK3β, and GSK3β **(c)**.

**Figure 3:**
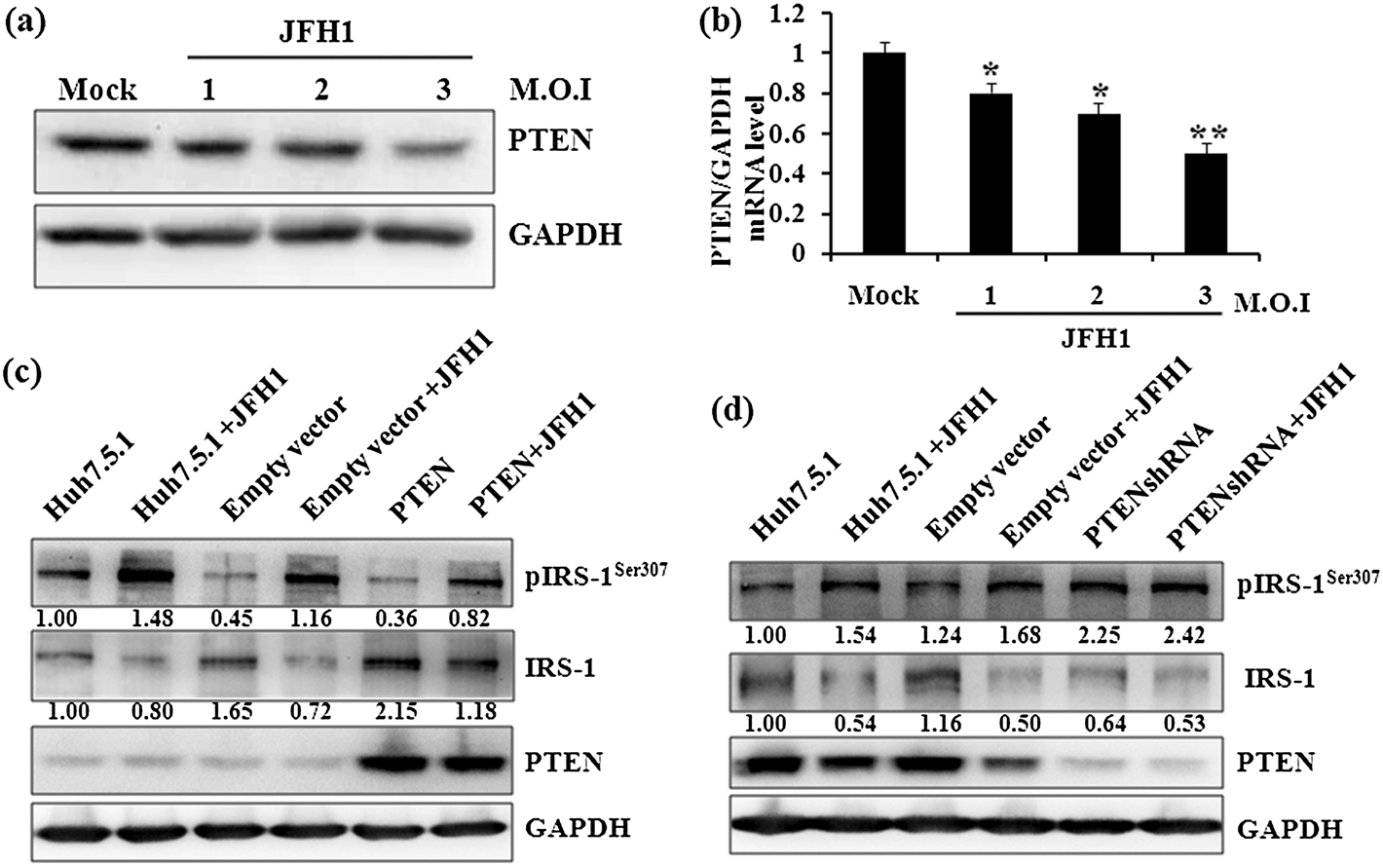
**Effect of HCV infection on PTEN expression in Huh7.5.1 cells.** Huh7.5.1 cells were routinely infected with different M.O.I of HCV 2a cc for 48 h and equal amounts of cellular lysates were subjected to western blotting for evaluating PTEN protein level **(a)** and qRT-PCR for PTEN mRNA expression **(b)**. Huh7.5.1 cells, empty lentivector groups, and PTEN or PTEN shRNA expressing cells were infected with HCV viral particles (M.O.I = 2). Representative immunoblots of pIRS-1^Ser307^, IRS-1, PTEN and GAPDH expression in cells showing PTEN overexpression **(c)** and downregulation **(d)**, respectively.

Because the PI3K/Akt signaling is involved in HCV-mediated IR, we next evaluated whether its negative regulator PTEN also altered its expression pattern during HCV infection. To elucidate this possibility, we detected PTEN expression in HCVcc-infected Huh7.5.1 cells at different multiplicity of infection (M.O.I) for 48 h by western blotting and real-time PCR (Figure 2). Compared with uninfected cells, HCV-infected cells showed a significant reduction in PTEN protein and mRNA expression levels (Figure 3a,b).

To further explore the impact of PTEN expression on the key effector IRS-1 that mediates IR during HCV infection, we examined pIRS-1 and IRS-1 levels in HCV-infected cells in which PTEN was either overexpressed or knocked down by the lentivirus. Compared to IRS-1 expression upon HCV infection in Huh7.5.1 cells and empty vector group, IRS-1 levels were partially rescued, and pIRS-1 levels were slightly reduced by PTEN overexpression regardless of JFH1-based HCVcc infection (Figure 3c). In contrast, IRS-1 levels were obviously decreased simply due to the depletion of PTEN (lanes 3 vs. 5 and lanes 4 vs. 6 in Figure 3d). This result suggested that pIRS-1 Ser307 might be another direct phosphorylational substrate for PTEN, since Yuji Shi et al. have demonstrated that PTEN is a protein tyrosine phosphatase for IRS-1 [16]. Moreover, phosphorylation of IRS-1 at Ser307 was considerably enhanced by PTEN knockdown, especially in the infected cells. Taken together, these data demonstrate that down-regulation of IRS-1 and increase of pIRS-1 at Ser307 upon HCV 2a infection occurs in a PTEN-dependent manner.

### HCV infection down-regulates PTEN expression via HCV core protein

**Figure 4:**
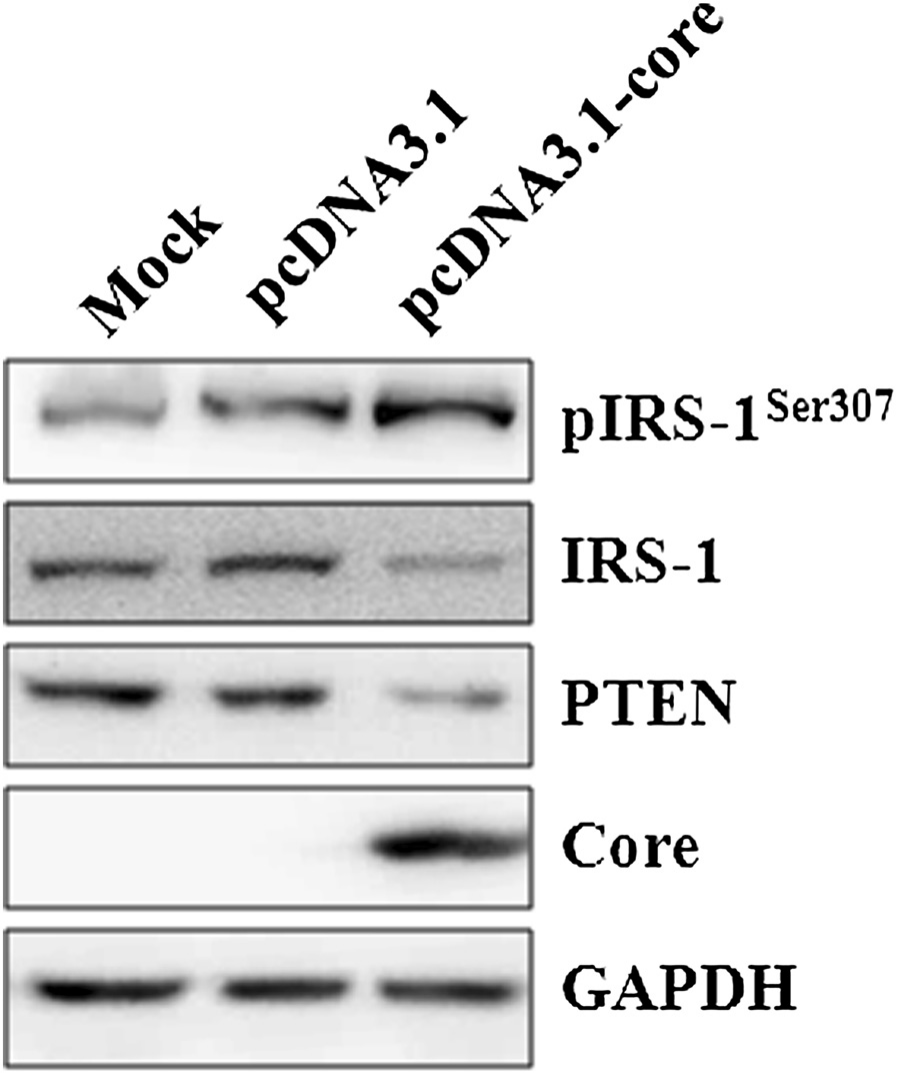
**Effect of transfection of Huh7.5.1 cells with JFH1-core plasmid on PTEN expression.** Huh7.5.1 cells transfected with empty vector or core expression vector were subjected to western blotting for pIRS1^Ser307^, IRS-1, PTEN and HCV core-specific antibodies. GAPDH was used as an internal control to verify protein loading in each well.

To elucidate the underlying mechanism by which HCV regulated PTEN and subsequent IRS-1 levels, Huh7.5.1 cells were transfected with one of the following plasmids: pc-JFH1-core expressing the HCV core protein from the strain JFH1 or pcDNA3.1 as the negative control (Figure 4). Our results showed that both PTEN and IRS-1 expression decreased, while IRS-1 Ser307 phosphorylation increased in the HCV core-transfected cells. These results indicate that HCV 2a core protein alone appears to be sufficient for repression of PTEN and IRS-1, as well as promotion of pIRS-1 at Ser307 in the insulin signaling pathway, which are molecular events linked with IR.

## Discussion

Our results demonstrate that HCV 2a infection interferes with the function of IRS-1 via down-regulation of PTEN expression and increase in phosphorylation of IRS-1 at Ser307 inHuh7.5.1 cells. This study uncovered a sequential event in which HCV regulates several critical insulin signaling molecules during its early infection, which partially contributes to the mechanical elucidation of HCV-associated IR.

Recently, chronic HCV infection has been regarded as an independent risk factor in the incidence of T2DM. Compared to HBV infection, the incidence of T2DM is approximately 1.8 to 2.5-fold higher in patients with chronic HCV infection. In such infected patients with no or minimal liver fibrosis, IR is the major factor that contributes to the occurrence of T2DM [17]. Therefore, there is a clinically relevant need to understand the explicit relationship between HCV infection and IR as well as the underlying mechanisms by which HCV perturbs insulin signaling pathway. On the basis of our study, we speculated that establishment of abnormal insulin signaling by HCV 2a should be the initial step that eventually leads to the HCV-associated IR.

PI3K/Akt signaling pathway is pivotal to the complex network of insulin signaling pathways. Activation of PI3K/Akt is a critical step to ensure subsequent phosphorylation of GSK3β and activation of glycogen synthase involved in insulin metabolic effects [15]. Association of insulin receptors with the IRS-1 adaptor protein upon insulin stimulation leads to phosphorylation and activation of downstream PI3K/Akt, and eventually promotes synthesis of glycogen and fatty acids to maintain normal blood glucose levels [18]. Our results suggest that HCV infection inhibits insulin-induced phosphorylation of Akt at Ser473 and subsequent phosphorylation of GSK3β (Figure 2a and c), suggesting that the decreased sensitivity to insulin results from the suppression of glucose uptake as well as glycogen synthesis. A previous study found instead that inhibition of phosphorylation at Thr-308 on Akt was more important than that at Ser473 for reduced insulin-stimulated glucose transport. This was thought to be due to the fact that phosphorylation of Akt at Ser473 appeared to precede phosphorylation by phosphoinositide dependent kinase-1 (PDK1) at Thr-308 [19]. Since we did not address phosphorylation of Akt at Thr308 in the present report, we cannot comment on its importance but can say definitively that impaired Ser473 phosphorylation is involved in reduced sensitivity to insulin during HCV infection.

Moreover, previous studies have indicated that HCV infection interferes with the insulin signaling cascade to promote IRS-1 and IRS-2 proteasomal degradation, by inducing the expression of SOCS3 and proinflammatory cytokines [10]. For example, the induction of TNF-α causes phosphorylation of IRS-1 at Ser312 and impedes insulin signaling, especially in HCV patients with more severe liver disorders [9,20]. It appears that different pathways targeted by different HCV genotypes perturb the normal insulin signaling pathway. In HCV 3a, HCV core protein promotes IRS-1 degradation via up-regulating suppressors of cytokine signaling 7 (SOCS7) and down-regulating peroxisome proliferator-activated receptor gamma (PPAR-γ). HCV 1b core protein impedes IRS-1 signaling through activation of the mammalian target of rapamycin (mTOR) activity [21]. A recent study suggested that HCV 2a suppresses IRS-1 function by activating the mTOR/S6K1 signaling pathway. It also perturbs glucose metabolism by down-regulating the glucose transporter type 4 (GLUT4) as well as up-regulating phosphoenolpyruvate carboxykinase 2 (PCK2) to promote IR [22,23].

It has been demonstrated that phosphorylation of IRS-1 at Ser1101 in the mTOR/S6K1 pathway makes IRS-1 releasing from intracellular complexes, leading to its degradation [24]. Likewise, in HCV-NS5A-expressing hepatoma cells, increased phosphorylation of IRS-1 at Ser307 was shown to be involved in enhanced gluconeogenic and lipogenic gene expression, thereby hampering metabolic activity and contributing to IR [25]. Therefore, phosphorylation of IRS-1 at Ser307 was further examined in our study, and we observed a similar increase of pIRS-1 at Ser307 with HCV 2a infection and core protein transfection (Figures 1 and 4). Our data further verifies that the increased pIRS-1 at Ser307 contributes to HCV-mediated IR.

As a negative regulator of the PI3K/Akt pathway, PTEN antagonizes insulin-dependent cell signaling, where its down-regulation improves insulin sensitivity. Del Campo et al. have suggested that HCV infection modifies the insulin signaling pathway by down-regulation of PTEN protein expression via post-transcriptional regulation events, especially in the presence of insulin [26]. Our results showed that HCV infection and core protein transfection down-regulated PTEN at both the protein and mRNA level. The data are somehow discrepant with those of a previous study wherein HCV 3a core protein triggered down-regulation of PTEN by interrupting its mRNA translation in a 3′-UTR-dependent manner [26,27]. The discrepancy between the two studies may due to different HCV genotypes. In addition, we also demonstrated that depletion of PTEN accelerated degradation of IRS-1 and promoted Ser307 phosphorylation of IRS-1, indicating that the balance between IRS-1 and pIRS-1 at Ser307 was PTEN-dependent.

Our results have revealed a central role for PTEN down-regulation in mediating the incidence of IR in HCV 2a-infected or pc-JFH-1-core plasmid transfected hepatocytes. The findings of our study support the recent view that alternation of PTEN expression/activity plays a crucial role in metabolic disorders of the liver [28]. Even Vinciguerra M et al. have suggested that liver-specific PTEN knockout mice were hypersensitive to insulin in spite of the development of steatosis and steatohepatitis [13], the role of PTEN in hepatic steatosis with IR, which is more common in HCV infection than in other chronic inflammatory liver diseases, is still a matter of debate.

Previous studies have suggested that IR in patients with chronic hepatitis C accelerates the process of liver disease and impaired sustained virological response to the standard therapy of peginterferon and ribavirin [7]. Thus, dysregulated PTEN expression caused by HCV infection may affect hepatic insulin sensitivity, but may also lead to progression to severe liver malignancies, of which PTEN is frequently mutated or deleted. Moreover, microRNAs, particularly miR-21, down-regulate PTEN to promote hepatocyte proliferation and invasiveness [29]. Indeed, strategies aimed to restore PTEN expression/activity with inhibition of IKK/NF-κB and mTOR signaling in injured hepatocytes might counteract hepatic IR, steatohepatitis and potentially more serious liver diseases for prolonged survival [14,30]. Recent studies by Serfaty L et al. have suggested that for the development of direct-acting antivirals (DAAs) such as telaprevir for administration to patients with chronic HCV genotype 1 infection, baseline Homeostasis Model Assessment on IR (HOMA-IR) was not a predictor for the achievement of sustained virological response (SVR) [31]. Nevertheless, DAA-based therapy is expensive and associated with many side effects. As long as peg-IFN/RBV is the standard therapy for chronic HCV infection, IR in HCV infection is worth noting. The detailed mechanisms by which HCV infection modifies the function of PTEN and the role of PTEN in the pathogenesis of HCV-associated IR and T2DM should be explored further.

Taken together, our results show that HCV infection triggers IR in a PTEN-dependent manner. The role of PTEN in HCV-associated IR is to promote the phosphorylation of IRS-1 at Ser307, resulting in the inhibition of the function of IRS-1. The findings provide new insight into the mechanisms of HCV-associated IR.

## Materials and methods

### Antibodies and reagents

Insulin and antibodies against IRS-1, pIRS-1 (Ser307), PTEN, Akt, pAkt (Ser473), GSK3β and pGSK3β (Ser9) were purchased from Beyotime, China. GAPDH antibody was from Abmart, Shanghai. HCV core monoclonal antibody was obtained from Abcam. Horseradish peroxidase (HRP)-conjugated secondary antibodies were from Santa Cruz Biotechnology.

### Cell culture

Human hepatoma Huh7.5.1 cells (kindly provided by Dr.Jing Zhong from Institute Pasteur of Shanghai, Chinese Academy of Sciences, Shanghai, China) were cultured in Dulbecco’s modified Eagle’s medium (DMEM) (Gibco BRL, USA) with 10% (v/v) fetal bovine serum (FBS) (Gibco) and 1 mM glutamine, 1% penicillin/streptomycin, 100 nM nonessential amino acids (NEAA) at 37°C in 5% CO_2_.

### Production of HCVcc genotype 2a (clone JFH1) and infectivity assays

The plasmid pJFH-1, as the temple for HCVcc generation, was kindly provided by T. Wakita (National Institute of Infectious Disease, Japan). Briefly, the pJFH-1 plasmid was linearized, transcribed with the MEGAscript T7 kit (Ambion, Austin, USA) in vitro and then delivered into Huh-7.5.1 cells by electroporation. Supernatants collected at 4 to 5 days post-transfection were centrifuged at 1000 rpm for 5 minutes and filtered through a 0.45 μm-pore-size cellulose acetate membrane (Nalgene, Rochester, NY). Virus particles were stored at −80°C.

At 2 days post infection, Huh7.5.1 cells were fixed and permeabilized with methanol, and then immunostained with serum from HCV patients. HCV-JFH1 titers were determined from cell culture supernatant by fluorescent focus-forming assay. The virus titer was calculated as focus-forming units/ml. In our study, the multiplicity of infection (M.O.I) for infection of hepatocytes was 1–2.

### Plasmid construction and transfection

The core coding regions of pJFH-1 plasmid were amplified using HS DNA Polymerase (Takara) with the primers as followed:

- forward: 5’-CGAATTCATGAGCACAAATCCTAAACCT-3’;
- reverse: 5’-GCTCTAGATTAGGCAGCAGAGACCGGAACGGTGATG-3’.

The fragment was subsequently subcloned into vector pcDNA3.1. The resulting constructs, the pc-JFH1-core plasmid was verified by sequencing and transiently transfected into Huh7.5.1 cells according to Lipofectamine 2000 (Inviteogen, Carlabsd, CA) instructions.

### Lentivirus of PTEN production

The human PTEN coding sequence was cloned into pRRLsin.PPTs.hCMV.GFPpre by PCR to generate pLenti-PTEN. The primer for PTEN amplification was

- forward: 5’- GCTCTAGAATGACAGCCATCATCAAAGAGA-3’;
- reverse: 5’- GCGTCGACTCAGACTTTTGTAATTTGTGTATGC-3’.

Then HEK 293 T cells were transient transfected with plasmid pLenti-PTEN, MDL, REV and VSVG to produce recombinant lentiviral particles. The supernatants were collected at 48 hours after transfection and then centrifugated at 3000 rpm for 5 min to remove cellular fragments. The collected lentiviral particles were then filtered through 0.45 μm pore-sized PVDF membranes and viral titers were calculated by quantitative realtime-PCR. Cells transducted with Lenti-EGFP were used as controls

To knockdown PTEN, we purchased GV248-shPTEN (short hairpin RNAs) targeting the following sequence 5’- ACAGCTAGAACTTATCAAA-3’ from Ji Kai Gene and verified by Western blot.

### Western blot assay

Cells were lysed with 0.1% SDS RIPA buffer (20 mM Tris–HCl, 250 mM NaCl, 3 mM EDTA, 10% glycerol (v/v)) (Beyotime) containing protease inhibitors and phosphates inhibitors on ice. Extracted proteins were quantitated by Bradford method (Beyotime) and then separated by SDS-PAGE (12.5% acrylaminde). Afterwards, proteins were transferred onto PVDF membranes (Millipore) and blocked in 5% non-fat milk for 2 h, and then incubated with primary antibodies overnight at 4°C, followed by the corresponding HRP-conjugated secondary antibodies for 1 h. The blots were finally detected by the enhanced chemiluminescence on Gene Gnome HR image capture (Cambridge, UK) and analyzed by GeneTools software. The primary antibodies used were anti-IRS-1, Akt, pAkt, PTEN, GSK3β, pGSK3β antibodies (1:500), HCV core (1:1000) and GAPDH antibody (1:2000).

### Reverse transcription and real-time PCR

Total cellular RNA was extracted by Trizol Reagent (Invitrogen) as instructions. 1 μg of total cellular RNA to cDNA synthesis was performed by M-MLV reverse transcriptase (Progema) with random hexamers. For the quantification of PTEN, semi-quantitative real-time PCR was performed with normalization for GAPDH using Uiversal SYBR Green Master (Takara) on a Rotor-Gene 3000 real-time thermal cycler (Corbett, Sydney, Australia). The following primers were used:

- PTEN,forward 5’-TTGAAGACCATAACCCACCA-3’
- and reverse 5’-CACATAGCGCCTCTGACTGG-3’;
- GAPDH,forward 5’-TGGGCTACACTGAGCACCAG-3’
- and reverse 5’-AAGTGGTCGTTGAGGGCAAT-3’.

All experiments were carried out in triplicate on a StepOne Plus real-time PCR system (Applied Biosystems) and the data was analyzed by SDS v2.3 software.

### Statistical analysis

Results were presented as mean ± standard deviation (SD) and analyzed by One-Way ANOVA test using SPSS software19.0. P <0.05 was considered statistically significant. All above experiments were performed at least three times.
